# Reasons for Reduced Moisture Resistance of Sulfur-Extended Asphalt Concrete

**DOI:** 10.3390/ma14237218

**Published:** 2021-11-26

**Authors:** Huu Tuan Le, Evgenij V. Korolev, Anna N. Grishina, Vitaly A. Gladkikh

**Affiliations:** REC “Nanomaterials and Nanotechnology”, Research Center “MGSU Stroy-Test”, National Research Moscow State University of Civil Engineering, 129337 Moscow, Russia; huutuan1511@gmail.com (H.T.L.); korolev@nocnt.ru (E.V.K.); grishinaan@mgsu.ru (A.N.G.)

**Keywords:** sulfur-bitumen binder, sulfur-bitumen paste, sulfur-extended asphalt concrete, moisture resistance, technical sulfur, chemical interaction of sulfur with mineral fillers, hydrogen sulfide, sulfur dioxide, sulfur-containing water-soluble calcium, and magnesium salts

## Abstract

The paper presents the results of a study of the mechanism for reducing the moisture resistance of sulfur-extended asphalt concrete. It is shown that a decrease in moisture resistance occurs due to the occurrence of chemical and physical processes. At the same time, it was found that during the manufacture of sulfur-bitumen composites, toxic gases H_2_S and SO_2_ are formed, which are capable of interacting with a mineral filler, as well as the interaction of sulfur with a mineral powder with the formation of sulfur-containing water-soluble salts, the extraction of which leads to a decrease in the moisture resistance of sulfur-bitumen materials. The change in the rate of leaching of these substances from the composite is due to the physical process caused by the crystallization of sulfur and the formation of a capillary structure, which significantly increases the rate of leaching of calcium and magnesium salts, which are products of dissolution or hydrolytic decomposition of water-soluble products of the interaction of sulfur, H_2_S and SO_2_ gases with calcium and magnesium carbonates. The intensity of chemical and physical processes intensifies with an increase in the amount of sulfur in sulfur-bitumen materials.

## 1. Introduction

The authors of works [1,2,3,4,5,6] have shown that the use of sulfur as a modifier for asphalt concrete is a promising direction, allowing the expansion of the temperature range of operation of asphalt concrete pavements in the area of high positive temperatures. In addition, in works [5,6,7] it is shown that the introduction of sulfur provides a decrease in the amount of bitumen in the asphalt concrete mixture, as well as a decrease in the temperature of preparation and laying The use of sulfur improves not only the operational properties of sulfur asphalt concrete (SEA) but also the technological properties of the mixture. Thus, in [8], the rheological properties of a bitumen-sulfur melt were investigated and it was shown that the introduction of sulfur significantly reduces the viscosity of such melts. Physicochemical processes of interaction of bitumen with sulfur are described in [9]. In which it is shown that the factor controlling the formation of H_2_S and SO_2_ is the concentration of hydrogen ions. In addition, the partial solubility of bitumen in the bitumen melt in an amount up to 10% of the amount of introduced sulfur has been established, the remaining sulfur forms a dispersed system such as an emulsion, in which the dispersed phase is molten sulfur droplets, and the dispersion medium is a melt of petroleum bitumen [10]. (Asphalt concrete with the addition of sulfur (technical or modified) received the name sulfur-extended asphalt concrete- SEA [10]). After cooling, crystallization of sulfur particles is observed, which increases the strength of sulfur asphalt concrete [11,12,13,14,15,16]. According to X-ray phase analysis, the formation of sulfur crystals is completed on the 7th day after the preparation of the material [17]. Further improvement of the technology of sulfur asphalt concrete is presented in [18]. Nevertheless, in works [19,20,21] it is shown that sulfur-asphalt concrete is characterized by insufficiently high water resistance with prolonged water saturation. The reasons for this may be [21]:

- Chemical interaction of sulfur and toxic gases formed during the interaction of sulfur with bitumen [21,22], with dispersed phases with the formation of water-soluble compounds (chemical hypothesis);
- The processes of crystallization of sulfur in the volume of the sulfur-bitumen binder and the formation of capillaries that increase the permeability of asphalt concrete for liquid media (physical hypothesis).

This article presents the results of assessing the possibility of implementing a chemical mechanism for reducing the water-resistance of sulfur asphalt concrete. The formulation of the formulated problem included the study of the recipe systems “oil bitumen—sulfur”, “sulfur—mineral filler”, and “oil bitumen–sulfur–mineral filler”.

## 2. Materials and Methods

The objects of research were selected: oil road bitumen, grade BND 60/90 (produced by Moscow Oil Refinery LLC, Moscow, Russia), which meets the requirements of state standard GOST 22245-90 “Road bitumen. Technical conditions”. The physical and mechanical characteristics of bitumen is presented in Table 1.

Non-activated dolomite mineral powder MP-1, complying with the requirements of GOST 52129-2003 “Mineral powder for asphalt concrete and organic-mineral mixtures. Technical conditions”. The chemical composition of dolomite powder is presented in Table 2.

Crystalline sulfur meeting the requirements of state standard GOST 127.1-93 “Technical sulfur. Technical conditions”. The main properties of technical sulfur are presented in Table 3.

Evaluation of the chemical interaction between sulfur and mineral fillers was carried out using sulfur mastics with a filling degree of 0.3. To study the kinetics of changes in the pH of the aqueous extract from sulfur-bitumen binders, sulfur-bitumen binders with a sulfur content of 20 were used; 30 and 40% by weight. To determine the kinetics of changes in the concentration of ions of a sample of sulfur with a mineral filler in the presence of bitumen (In accordance with the accepted terminology, it is advisable to call such materials sulfur-bitumen paste (hereinafter referred to as SBP).), we used sulfur-bitumen paste with a sulfur content of 20; 30 and 40% by weight.

The study of the processes of structure formation and properties was carried out using an X-ray diffractometer ARL X’tra in the range of angles 2θ from 4° to 70°, in a step-by-step mode (0.02°) with an exposure duration at a point of 1 sec; FTIR-spectrometer Cary 630 (Agilent Technologies Inc., California, United States); pH meter pH/Ion Meter 781 (METROHM, Herisau, Switzerland); the content of calcium and magnesium ions was carried out by the titrimetric method. Determination of moisture resistance of sulfur mastics was carried out in accordance with GOST 25246-82- Chemically resistant concrete.

## 3. Results and Discussion

**Figure 1:**
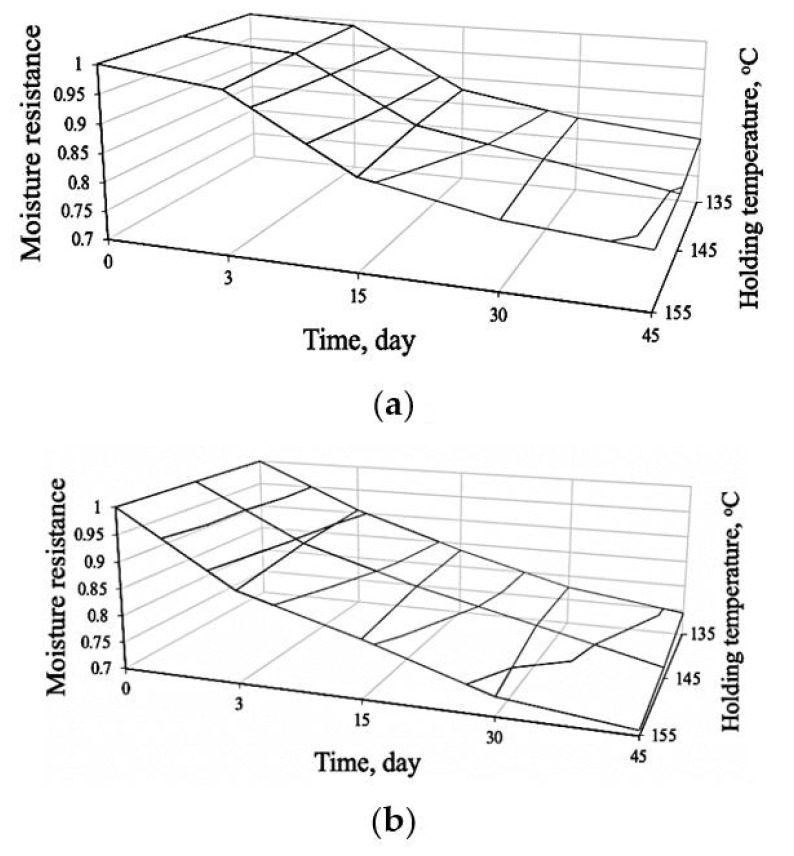
Kinetics of change in the coefficient of moisture resistance of sulfur mastics: (**a**) filler—mineral powder MP-1; (**b**) filler—fly ash.

It was shown in [23] that water-soluble compounds can form at the interface between sulfur and mineral filler. Water penetrating into the bulk of the material, by dissolving these compounds, violates the strength of the contact, which naturally leads to a decrease in the moisture resistance of the material. In this regard, the assessment of the chemical interaction between sulfur and mineral powder was carried out according to the kinetics of changes in the moisture resistance of sulfur mastics—composite materials consisting of sulfur, mineral filler, and additives to regulate the desired properties. The samples were made at temperatures of 135; 145 and 155 °C. The duration of the isothermal holding of the mixture was 3 h. The research results are shown in Figure 1.

The presented experimental data can be described by the classical kinetic equation [24]:

(1) $$y\left(t\right)=y_{m}\left(1-exp\left(-\alpha t^{n}\right)\right),$$

where *y_m_*–the limiting value of the investigated material characteristics; *α*, *n*–constants determined from experimental data; *t*–time.

The constant *α* and *n* in formula (1) for describing the data in Figure 1 depend on temperature and, in the first approximation, can be described:

(2) $$y\left(t\right)=y_{m}\left(1-exp\left(-\alpha t^{n}\right)\right),$$

where *T*–temperature of manufacture of sulfur mastics; *a*, *b*, *c*–empirical coefficients, the values of which are given in Table 4.

Taking into account formula (2) and $k_{ct,m}=1$, dependence (1) can be transformed to the form:

(3) $$k_{ct}\left(t\right)=1-exp\left(-\alpha \left(T\right)t^{n\left(T\right)}\right).$$

Analysis of Table 4 shows the presence of a significant effect on the chemical composition of the filler. Thus, *α* = *f(T)* is a coefficient characterizing the intensity of a decrease in the resistance coefficient—for sulfur mastics based on MP-1 it has a descending character, and for sulfur mastics based on fly ash it is ascending. Moreover, the intensity of change *α* = *f(T)* for sulfur mastic on fly ash, is significantly higher. The coefficient *n* = *f(T)* for the considered compositions of sulfur mastics increases in modulus with increasing temperature for sulfur mastics on fly ash much more intensively.

It is shown in [23] that the decrease in the moisture resistance of sulfur mastics made from fly ash is explained by the formation of water-soluble silicon sulfide at the interface between sulfur and fly ash. For sulfur mastics on MP-1, the reason for the decrease in moisture resistance requires clarification.

**Figure 2:**
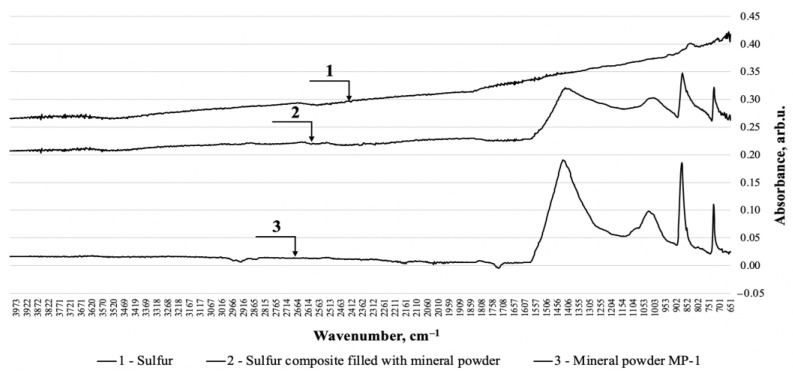
FTIR-spectrogram of a sulfur composite filled with mineral powder MP-1.

Studies of sulfur mastics on MP1 are shown in Figure 2. The IK spectrum of the mineral powder MP1 shows anomalies at wavenumbers: 1417; 874 and 727 cm^−1^ refer to vibrations in the CO_3_-group in calcite crystals, and at 1007 cm^−1^—to the CO_3_-group in crystals of a variety of calcium carbonate—aragonite [25]. In the IK spectrum of sulfur, characteristic maxima are not shown, since the wavenumbers for sulfur are in the range of 450 to 600 cm^−1^. On the IK spectrogram of the sulfur composite, only the maxima corresponding to the anomalies’ characteristic of the mineral filler MP1 are identified; in the investigated range of wavenumbers, there are no new responses, which indicates the absence of chemical bonds that vibrate under the influence of the investigated wavelengths.

**Figure 3:**
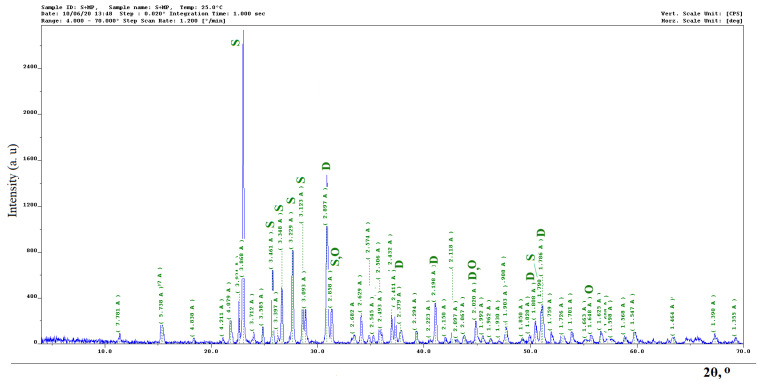
X-ray diffraction pattern of a sulfur composite filled with mineral powder MP-1: S–sulfur; D–dolomite; O–oldhamite.

On a typical radiograph of a sulfur composite (Figure 3), the main maxima characteristic of sulfur is revealed (3.86; 3.46; 3.23; 3.34; 3.12; 2.86; 1,79 Å) and mineral powder MP1, represented by dolomite (2.90; 2.20; 1.81; 1.79; 2.02; 2.41 Å). In addition, the X-ray revealed a new phase–oldhamite (2.86; 2.02; 1.65 Å), which is the product of the chemical interaction of mineral powder MP1 and sulfur. Additional studies are required to determine the phases related to unidentified maxima. This is due to the limited sensitivity of the X-ray phase analysis method in identifying individual phases [26].

**Figure 4:**
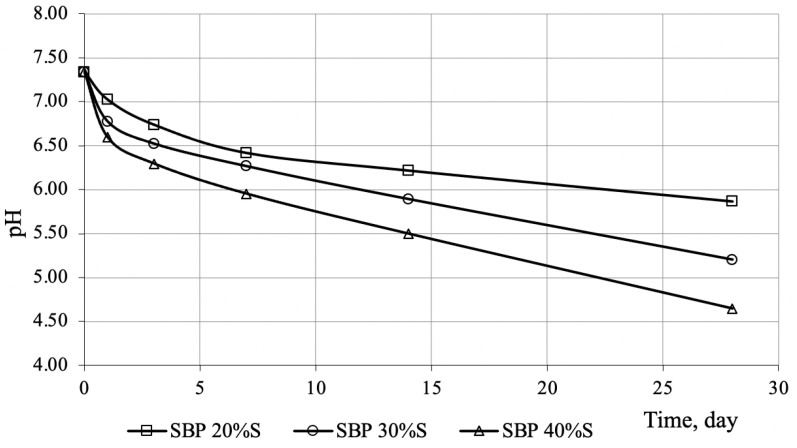
Kinetics of pH change in the aqueous extract from sulfur-bitumen binders.

The reasons for the formation of these water-soluble products are both the chemical interaction of sulfur itself and the toxic gases H_2_S and SO_2_ with mineral powder. To assess the contribution of toxic gases to the formation of water-soluble substances, it is necessary to determine their residual concentration in the sulfur-bitumen binder (hereinafter–SBB). For this, it is necessary to study the kinetics of changes in the pH of water upon exposure of samples of a sulfur-bitumen binder to it. Obviously, when exposed to water, pH will change both when H_2_S and SO_2_, formed during the interaction of sulfur and bitumen components, are dissolved in it, and gases contained in the initial components. Therefore, this factor was taken into account when processing the results. The kinetics of the change in the pH of sulfur-bituminous binders is shown in Figure 4.

The data in Figure 4 demonstrate a decrease in pH, and the intensity of this process depends on the sulfur content: the greatest intensity of the decrease in pH (by 37%) is observed for SBB containing 40% sulfur. This indicates the presence of residual amounts of H_2_S and SO_2_, which can participate in chemical interaction with the filler. This, in particular, is evidenced by the data [9] on the emission of these gases from sulfur-bitumen mixtures.

The presence of water-soluble compounds formed during the interaction of sulfur or H_2_S and SO_2_ with a mineral filler in the presence of bitumen can be identified by the kinetics of changes in the concentration of calcium and magnesium ions in the aqueous extract. The scientific basis of this method is the assumption of an increase in the concentration of calcium and magnesium ions in the water extract as a result of dissolution or hydrolytic decomposition of water-soluble products of the interaction of sulfur, gases H_2_S and SO_2_ with calcium and magnesium carbonates. It is advisable to evaluate low concentrations of calcium and magnesium ions in terms of the hardness of the water extract since the method allows for high accuracy in determining low ion concentrations due to the characteristics of soluble substances. In addition to these products of chemical interaction, the concentration of calcium and magnesium ions in the solution will increase due to the dissolution of the mineral powder in it. Therefore, to establish the presence of water-soluble sulfur-containing salts of calcium and magnesium, it is necessary to analyze a control composition that does not contain sulfur.

Hence, it follows that when the test samples are kept in water, the concentration of calcium and magnesium ions, which determine the hardness of the water, will increase in the presence of sulfides of these metals in the material. Moreover, it is significantly more intense compared to the control composition that does not contain sulfur.

**Figure 5:**
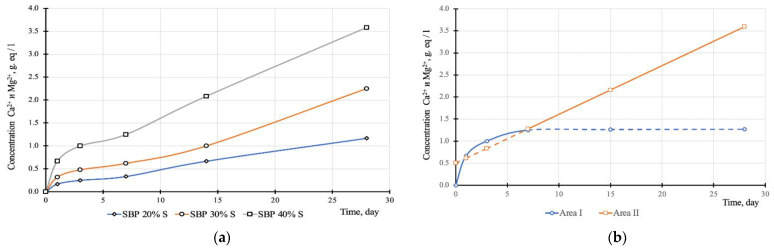
Kinetics of changes in the concentration of calcium and magnesium ions in the sulfur bitumen paste (**a**) kinetics of changes in the concentration of calcium and magnesium ions in the sulfur bitumen paste with a sulfur content of 20; 30 and 40% by weight; (**b**) two characteristic areas on the kinetic curves: 0–7 days and 7–28 days.

The results of experimental studies are presented in Figure 5, the analysis of which demonstrates that two characteristic areas can be distinguished on the kinetic curves: 0–7 days and 7–28 days (Figure 5b). Moreover, in these areas, the kinetics of changes in the concentration of calcium and magnesium ions in the water extract is fundamentally different:

- In the first section, a classical exponential increase in concentration is observed, which clearly correlates with the depth of water penetration into the volume of the material sample and can be described by the function (1);
- In the second section, a linear increase in concentration is observed, which can only be explained by a significant increase in the permeability of the material.

The values of the empirical coefficients of function (1) for the first section of the kinetics and the linear equation (*C(t)* = *b* + *kt*, where *C(t)* is the concentration of calcium and magnesium ions in the aqueous extract; *k*, *b* are empirical coefficients) for the second section are given in Table 5.

Analysis of the data in Table 5 shows that in the first section, the dependences of the coefficients *C_max_ = f(S)*, *α = f(S)*, and *n = f(S)* on the sulfur content in SBB are linear. It is shown in [9] that sulfur in a sulfur-bitumen binder gradually crystallizes from the moment of manufacture, reaching the maximum degree of crystallization on the 5th day. Probably, the crystallization of sulfur leads to the formation of a capillary structure in the volume of bitumen, which, due to its high viscosity, persists for a period of time sufficient for filling with water. These capillaries perform a transport function, intensifying metabolic processes. It is natural that the number of such capillaries increases with an increase in the sulfur content, therefore, in the second section of the *C = f(t)* dependence, there is a clear relationship between the sulfur content in the SBP and an increase in the concentration of calcium and magnesium ions in the water extract, which can be characterized by the coefficient *k* (Table 5). Thus, the presented data show that in the process of manufacturing a sulfur-bitumen binder, sulfur interacts with a mineral filler with the formation of water-soluble sulfur-containing calcium and magnesium salts. In addition, due to the crystallization of sulfur, the permeability of the material increases, which leads to an intensification of the extraction process of calcium and magnesium ions. These processes naturally increase with an increase in the sulfur content in the SBP.

It is natural to assume that on the kinetic dependences *pH = f(t)* (Figure 4), the same areas with coincident boundaries should be distinguished. The empirical coefficients of the functions, taking into account the described physical quantity for the first and second sections, are presented in Table 6, the analysis of the data of which demonstrates the similarity of the dependence of the empirical coefficients on the sulfur content and the coincidence of the location of the selected sections.

Removal of water-soluble products of interaction between sulfur and mineral powder from the composite during its operation leads to a violation of the integrity of the contact at the border “sulfur-bitumen binder—mineral filler”, and, consequently, to a decrease in the contact area at this border. This naturally leads to a decrease in the strength of the material.

**Figure 6:**
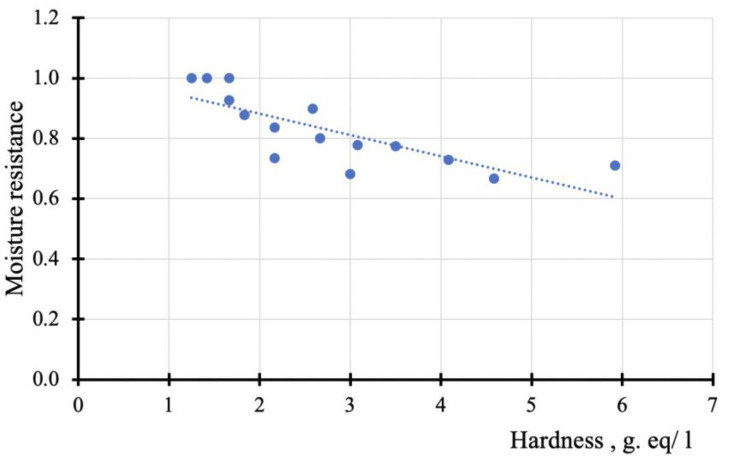
Correlation between the concentration of calcium and magnesium ions and the moisture resistance of SBP.

The conducted studies suggest that there should be a correlation between the coefficient of moisture resistance and the value characterizing the degree of destruction of the contact area—the value of the hardness of the water extract (Figure 6).

According to Figure 6, the correlation coefficient between the coefficient of moisture resistance and the concentration of calcium and magnesium ions in water extracts during exposure of SBP samples has a corresponding orientation and a high-value *r* = −0.82.

## 4. Conclusions

Thus, it was found that in the system “bitumen–sulfur–mineral powder” chemical and physical processes occur, leading to a decrease in the moisture resistance of sulfur-bitumen materials. The chemical process in the temperature range for the preparation of sulfur-bitumen materials is due to the interaction of sulfur with bitumen with the formation of H_2_S and SO_2_, which are capable of interacting with a mineral filler. Additionally, the process of interaction of sulfur with a mineral filler takes place. These processes of chemical interaction led to the formation of sulfur-containing salts of calcium and magnesium. The physical process is due to the process of crystallization of sulfur with the formation of a capillary structure in sulfur-bitumen materials and the process of dissolution of water-soluble sulfur-containing salts of calcium and magnesium. The capillary structure performs a transport function, increasing the access of water to the contact boundary “sulfur-bitumen binder–mineral filler” and the extraction of calcium and magnesium ions, which are the products of dissolution or hydrolytic decomposition of water-soluble products of the interaction of sulfur, gases H_2_S and SO_2_ with calcium and magnesium carbonates. The intensity of chemical and physical processes intensifies with an increase in the amount of sulfur in sulfur-bitumen materials.

Removal of water-soluble sulfur-containing salts of calcium and magnesium leads to a natural decrease in the moisture resistance of sulfur-bitumen materials. The hardness of water extracts during exposure to sulfur-bitumen materials can be an indicator for optimizing the production technology of such materials and developing ways to increase their water resistance.

## Figures and Tables

**Table 1:** The physical and mechanical characteristics of bitumen.

Index	Actual Values
Penetration depth of the needle at 25 °C, 0.1 mm	69
Depth of needle penetration at 0 °C, 0.1 mm	34
Elongation at 20 °C, cm	82.7
Elongation at 0 °C, cm	3.7
Softening point, °C	53
Brittleness temperature, °C	−20
Change in softening temperature after heating, °C	5
Penetration index	−0.6
Flash point, °C	254

**Table 2:** The chemical composition of dolomite powder.

Material	Content, % by Weight			
	SiO_2_	Al_2_O_3_	Fe_2_O_3_	CaCO_3_ + MgCO_3_
Dolomite	7.64	0.34	1.12	90.9

**Table 3:** The main properties of technical sulfur.

Indicator Name	Actual Values
Appearance	yellow granules
True density, g/cm^3^	2.07
Bulk density, g/cm^3^	1.05
Mechanical pollution (paper, wood, sand, etc.)	absent

**Table 4:** Values of empirical coefficients. (*α* = *f*(*T*) and *n* = *f*(*T*)).

Composition	Dependence	Coefficient Values		
		*a*	*b*	*c*
Sulfuric mastic for MP-1	α=f(T)	−6.85	0.1515	−0.0006
	n=f(T)	−0.39	0.0032	−0.00001
Sulfuric mastic on fly ash	α=f(T)	40.03	−0.5333	0.0019
	n=f(T)	−6.43	0.0895	−0.0003

**Table 5:** Empirical coefficients of the kinetics of changes in the concentration of calcium and magnesium ions in the water extract in the study of SBP samples.

**Sulfur Content in SBB, %**	Empirical Coefficients				
	Area I			Area II	
	$C_{max}\;$	α	n	k	b
20	0.34	0.67	0.62	0.039	0.083
30	0.64	0.71	0.64	0.079	0.005
40	1.26	0.75	0.67	0.111	0.500

**Table 6:** Empirical coefficients of the kinetics of changes in the pH of the aqueous extract from sulfur-bitumen binders.

**Sulfur Content in SBB, %**	Empirical Coefficients				
	Area I			Area II	
	$pH_{min}$	n	α	k	b
20	6.21	0.18	2.04	−0.026	6.59
30	6.10	0.18	2.22	−0.050	6.56
40	5.95	0.23	2.22	−0.062	6.38
